# Biofilm formation inhibition and dispersal of multi-species communities containing ammonia-oxidising bacteria

**DOI:** 10.1038/s41522-019-0095-4

**Published:** 2019-08-27

**Authors:** Pejhman Keshvardoust, Vanessa A. A. Huron, Matthew Clemson, Florentin Constancias, Nicolas Barraud, Scott A. Rice

**Affiliations:** 1 The School of Biotechnology and Biomolecular Sciences, UNSversatile open source tool for metagenomicsW Sydney, Sydney, NSW Australia; 2 The Centre for Marine Bio-Innovation, UNSW Sydney, Sydney, NSW Australia; 3 Rural Clinical School, UNSW Sydney, Sydney, NSW Australia; 4 The Singapore Centre for Environmental Life Sciences Engineering, Nanyang Technological University, Singapore, Singapore; 5 Genetics of Biofilms Unit, Institut Pasteur, 25-28 Rue de Dr Roux, 75015 Paris, France; 6 The School of Biological Sciences, Nanyang Technological University, Singapore, Singapore; 7 Ithree Institute, University of Technology Sydney, UTS Faculty of Science Store, Building 1, Level 2, Thomas Street, Ultimo, NSW 2007 Australia

**Keywords:** Biofilms, Microbial ecology

## Abstract

Despite considerable research, the biofilm-forming capabilities of *Nitrosomonas europaea* are poorly understood for both mono and mixed-species communities. This study combined biofilm assays and molecular techniques to demonstrate that *N*. *europaea* makes very little biofilm on its own, and relies on the activity of associated heterotrophic bacteria to establish a biofilm. However, *N*. *europaea* has a vital role in the proliferation of mixed-species communities under carbon-limited conditions, such as in drinking water distribution systems, through the provision of organic carbon via ammonia oxidation. Results show that the addition of nitrification inhibitors to mixed-species nitrifying cultures under carbon-limited conditions disrupted biofilm formation and caused the dispersal of pre-formed biofilms. This dispersal effect was not observed when an organic carbon source, glucose, was included in the medium. Interestingly, inhibition of nitrification activity of these mixed-species biofilms in the presence of added glucose resulted in increased total biofilm formation compared to controls without the addition of nitrification inhibitors, or with only glucose added. This suggests that active AOB partially suppress or limit the overall growth of the heterotrophic bacteria. The experimental model developed here provides evidence that ammonia-oxidising bacteria (AOB) are involved in both the formation and maintenance of multi-species biofilm communities. The results demonstrate that the activity of the AOB not only support the growth and biofilm formation of heterotrophic bacteria by providing organic carbon, but also restrict and limit total biomass in mixed community systems.

## Introduction

Nitrifying organisms, such as ammonia-oxidising bacteria (AOB) and nitrite-oxidising bacteria, are found globally in environments ranging from soil^1^ to oceans.^2^ They are vital to nitrogen cycling, and are used extensively in wastewater treatment, typically as biofilms,^3,4^ to remove nitrogenous compounds such as ammonia and nitrite. Research in nitrification has focused on *Nitrosomonas europaea* as a model AOB, and has linked traits, such as ammonia oxidation efficiency and stress resistance to the ability of *N*. *europaea* to form biofilms.^5,6^

*N*. *europaea* is a slow or poor biofilm former, typically requiring several weeks to achieve a robust, functional biofilm on surfaces, such as in drip-flow reactors,^5^ on slides or other support matrices.^7^ *N. europaea* produces an extracellular matrix of extrapolymeric substances, composed partly of extracellular DNA (eDNA), that contribute to the formation and three-dimensional structure of biofilms.^8^ *N*. *europaea* also encodes putative capsular polysaccharide biosynthesis genes,^9^ suggesting that polysaccharides and eDNA may be the primary constituents of the biofilm matrix.

The relatively long generation time of *N*. *europaea* cells in pure culture starkly contrasts with the more rapid development of nitrifying biofilms in fast-flowing drinking water systems, which led to the suggestion that *N*. *europaea* may form synergistic relationships with other organisms that are more adept at forming biofilms.^10^ The long generation time of *N*. *europaea* is commonly linked to the low-energy yield from ammonia oxidation, with approximately 65% of the energy from ammonia oxidation being used for cell maintenance activities, such as carbon fixation and regeneration of the quinolone pool used in ammonia oxidation, rather than for growth and biofilm development.^11^

Mixed-species bacterial biofilm communities have been shown to exhibit synergistic behaviour, including enhanced biofilm biomass generation,^12,13^ elevated resistance to antimicrobial compounds^13–15^ and co-metabolism of recalcitrant compounds.^16^ Thus, given the slow metabolism and limited biofilm formation of AOB, it has been suggested that heterotrophic bacteria may contribute to enhance biofilm formation by these bacteria.^17^ In exchange, heterotrophic bacteria such as *Pseudomonas spp*., which require organic carbon compounds for metabolism, may benefit from the secreted or released metabolic by-products of AOB, such as citrate,^9^ that could provide sufficient nutrients to support their growth in mixed community biofilms with nitrifying bacteria.^18^ A recent study by Petrovich et al.^19^ demonstrated enhanced biofilm formation upon incubation of *Pseudomonas* *aeruginosa* and *N*. *europaea* in continuous culture with nutrient-rich media. That study established a role for functionally distinct species in mixed consortia, and both organisms were provided with nutrients suitable for each species. However, drinking water distribution systems typically aim to minimise concentrations of available organic carbon,^20,21^ and so it is necessary to explore these relationships under conditions where organic carbon is a limiting factor in addition to the carbon-replete conditions used by Petrovich et al. (2017). What remains to be understood is how metabolic interdependence of such mixed communities would influence community formation, and the formation of biofilms.

It is hypothesised here that in addition to enhancing existing biofilms in single-species cultures, nitrifying bacteria play a role in mixed-species biofilm formation under variable nutrient conditions through the provision of accessible nutrients for heterotrophic bacteria via the turnover of organic compounds after chemoautotrophic oxidation of ammonia. In return, heterotrophic bacteria then readily produce extracellular biofilm components, such as polysaccharides, which may facilitate the establishment of AOB within biofilms.

To test these hypotheses, microbial biomass and community composition profiles were determined in the presence and absence of glucose and/or nitrification inhibitors. This work investigated the effect of inhibiting nitrification on both developing and established mixed-species nitrifying biofilms, to enhance our understanding of the role of ammonia-oxidising bacteria in the formation and maintenance of these biofilms. In particular, the interaction between AOB and heterotrophic bacteria under varying conditions of nutrient availability was investigated using a mixed-species culture containing *N*. *europaea* and a range of heterotrophic bacteria, including Pseudomonadaceae and Vibrionacaea, as well as others. The results suggest that AOB are vital for the formation of mixed-species biofilms under the carbon-limited conditions found in treated drinking water, and that the role of AOB extends beyond that of the provision of organic carbon, by regulating the size of the biofilm in response to biological cues.

## Results

### Biofilm formation by nitrifying bacterial communities with inorganic carbon as the sole carbon source

**Fig. 1:**
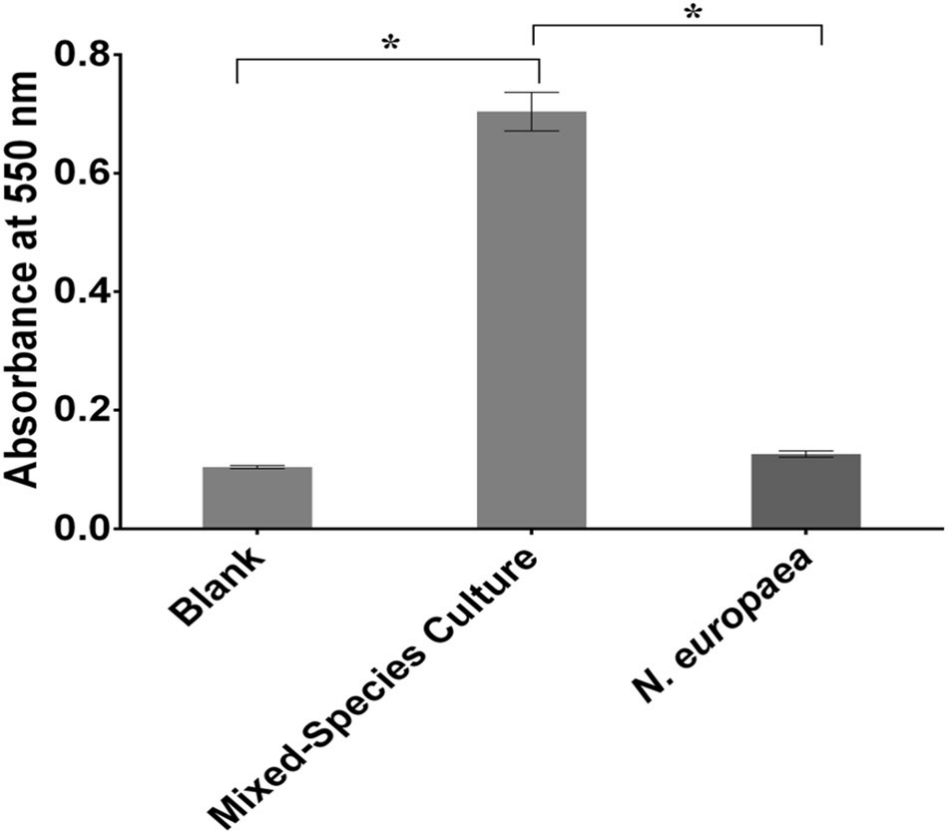
Differences in biofilm formation by a pure culture of *N*. *europaea* and a mixed-species nitrifying culture containing *N*. *europaea* and heterotrophic bacteria. Bacteria were grown in Nitrification medium and biofilm attachment was measured after 3 days using crystal violet staining, with sterile Nitrification medium serving as the blank control. Error bars represent the SEM (*n* = 6). Statistical significance, *p* < 0.05, is indicated by *

The ability of nitrifying bacteria to form biofilms was first assessed in culture conditions with inorganic carbonate ions as the sole carbon source with either (a) a pure culture of the AOB, *N*. *europaea* or (b) a mixed-species culture containing *N*. *europaea* and heterotrophic bacteria. Under conditions in which inorganic carbonate ions were the sole carbon source, the mixed-species community formed significantly more biomass (OD_550_ ~0.70, *p* < 0.05, *n* = 6), as determined by increased crystal violet (CV) staining, compared to the pure *N*. *europaea* culture (OD_550_ ~0.13). The wells containing the AOB were indistinguishable from the uninoculated nitrification medium serving as the control at 4 days (OD_550_ ~0.10) (Fig. 1), showing that *N*. *europaea* does not form any appreciable biofilm under these conditions.

### Inhibition of biofilm formation by a mixed-species nitrifying community

**Fig. 2:**
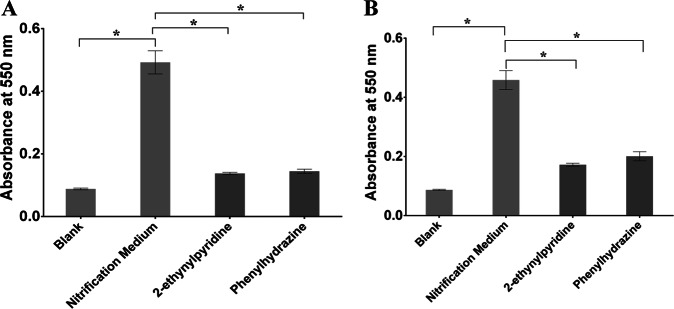
Effect of nitrification inhibitors on biofilms of a mixed-species nitrifying community. **a** For the prevention of biofilm development, cells were grown in Nitrification medium with the nitrification inhibitors (100 µM 2-ethynylpyridine or 1 µM phenylhydrazine) for 4 days; **b** For biofilm dispersal, biofilms were first formed for 3 days and were then exposed to the inhibitors for 24 h. For both conditions, biofilm biomass was measured using crystal violet staining. Error bars represent the SEM (*n* = 3). Statistical significance, *p* < 0.05, is indicated by *

To determine the role of AOB in the formation of mixed-species biofilms, nitrification inhibitors were added to the mixed-species nitrifying cultures. The addition of 2-ethynylpyridine (ammonia monooxygenase (AMO) inhibitor) (OD_550_ ~ 0.14) or phenylhydrazine (hydroxylamine oxidoreductase (HAO) inhibitor) (OD_550_ ~ 0.14), at concentrations known to block nitrite production, significantly reduced biofilm formation in the mixed-species communities, by approximately 70%, as determined by CV staining (Fig. 2a). The absorbance values for these treated biofilms were indistinguishable from the sterile nitrification medium acting as a control (OD_550_ ~ 0.09).

### Inhibition of AOB activity induces dispersal of mixed-species nitrifying biofilms

It has previously been shown that changes in nutrient concentration, such as the decreased availability of a carbon source,^22^ can induce biofilm dispersal. As shown above, biofilm formation was inhibited when the mixed-species culture was grown in the presence of the nitrification inhibitors, 2-ethynylpyridine or phenylhydrazine. Given that these compounds inhibit the ability of the AOB to generate organic carbon, the addition of the nitrification inhibitors would block the subsequent production of the organic carbon required to support continued growth of the heterotrophs. Therefore, it was of interest to determine if nitrification inhibitors could induce dispersal in in biofilms that had already formed, rather than only inhibiting their formation.

Biofilms of nitrifying communities were established in nitrification medium for 3 days before exposure to 2-ethynylpyridine or phenylhydrazine. After 24 h, the biofilms were significantly reduced by 2-ethynylpyridine (OD_550_ ~ 0.17, *p* < 0.05, *n* = 3) or phenylhydrazine (OD_550_ ~ 0.2, *p* < 0.05, *n* = 3), representing a reduction of 63% and 57%, respectively, compared to the untreated controls (OD_550_ ~ 0.46) (Fig. 2b). Interestingly, 2-ethynylpyridine, which induces complete inhibition of the nitrification pathway, and phenylhydrazine, which induces a partial inhibition of the nitrification pathway, both dispersed biofilms to similar extents (*p* > 0.05, *n* = 3), suggesting that a fully active nitrification pathway is required to maintain these biofilm communities. Collectively, these data suggest AOB and heterotrophic bacteria interact positively to form and maintain biofilms when inorganic carbon is present as the sole carbon source.

### The effect of glucose supplementation on the formation and composition of mixed-species nitrifying biofilms

**Fig. 3:**
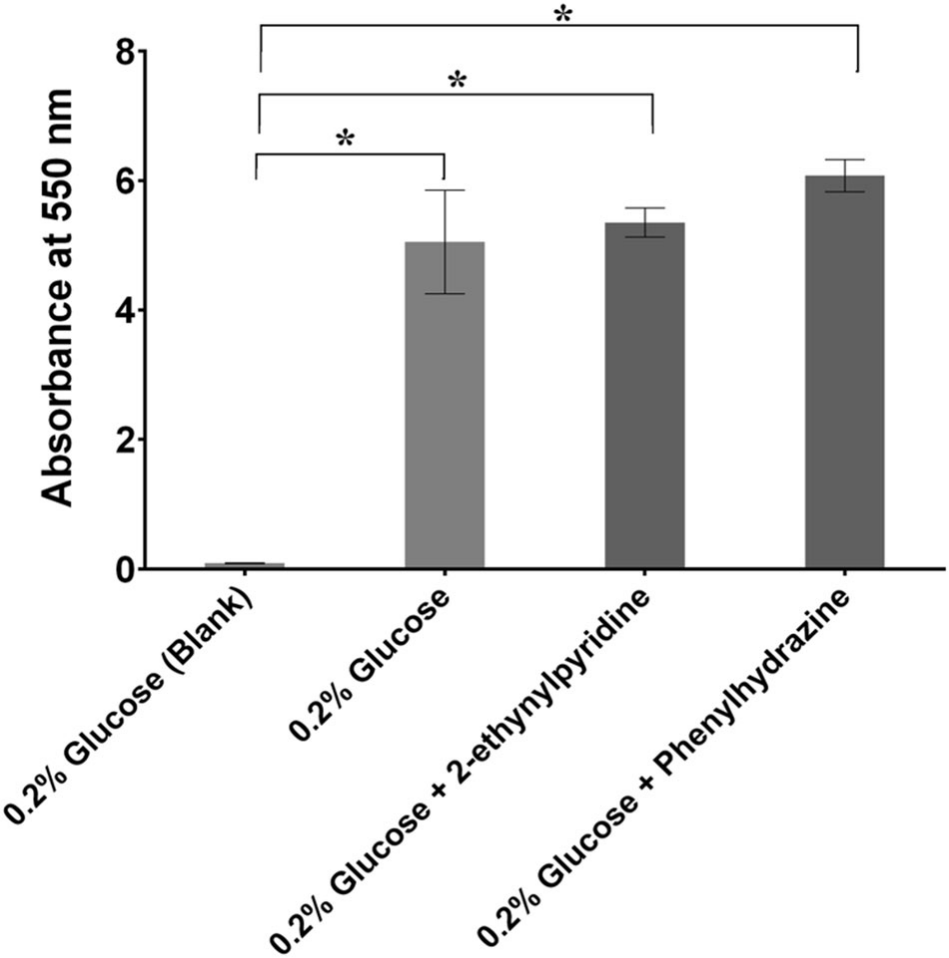
The effect of glucose on biofilm formation of the mixed-species biofilm. Bacteria were grown in nitrification medium supplemented with 0.2% glucose (w/v), with or without 100 µM 2-ethynylpyridine or 1 µM phenylhydrazine, in a 12-well microtitre plate for 3 days. Biomass was measured using crystal violet staining and error bars represent SEM (*n* = 3). Sterile nitrification medium with 0.2% glucose (w/v) served as the blank control. Statistical significance, *p* < 0.05, is indicated by *

As shown above, mixed-species biofilm development is inhibited or dispersed in response to the inhibition of nitrification when inorganic carbon is the only carbon source present. To further explore the role of nutrients in mediating mixed-species interaction, a mixed-species nitrifying community was supplemented with 0.2% glucose (w/v) as an alternative carbon source, which would bypass the need for the AOB to provide organic carbon to the heterotrophic bacteria. The cultures formed significantly more biomass (OD_550_ ~5.05, *p* < 0.05, *n* = 3) (Fig. 3), as determined by increased CV staining, when grown in Nitrification medium supplemented with 0.2% glucose (w/v) compared to the nitrification medium lacking an organic carbon source (OD_550_ ~0.70) (Fig. 2b). When also treated with 2-ethynylpyridine or phenylhydrazine in addition to supplementation with glucose, the biomass was not significantly affected by inclusion of the nitrification inhibitors, 2-ethynylpyridine (AMO inhibitor) (OD_550_ ~ 5.35, *p* > 0.05, *n* = 3) and phenylhydrazine (HAO inhibitor) (OD_550_ ~ 6.08, *p* > 0.05, *n* = 3), demonstrating that these compounds do not inhibit the heterotrophic bacteria in the community.

**Fig. 4:**
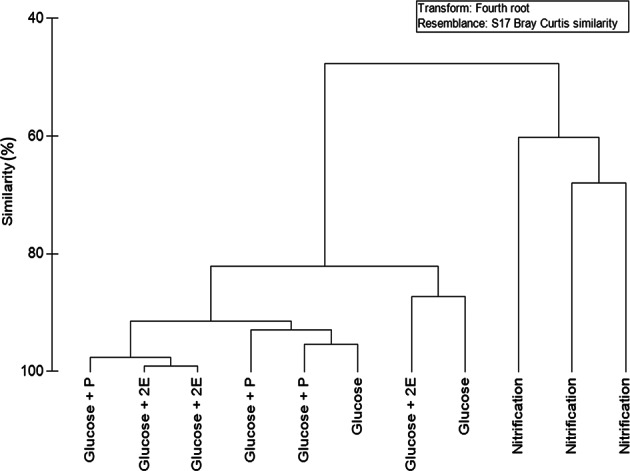
The effect of glucose supplementation on community composition and in a 4 days biofilm of a mixed-species nitrifying community. Cells were grown in Nitrification medium (nitrification) or nitrification medium supplemented with 0.2% glucose (w/v) (Glucose), with or without 100 µM 2-ethynylpyridine (2E) or 1 µM phenylhydrazine (P). The community composition was determined by LH-PCR (*n* = 3) and processed into a similarity table using PRIMER-6 software

Length heterogeneity polymerase chain reaction (PCR) (LH-PCR) was used to determine differences in the effect of glucose supplementation and nitrification inhibition on the diversity of the mixed-species nitrifying community when added at the point of inoculation or after establishment of the biofilm. The community composition of glucose-supplemented biofilms was not affected by the nitrification inhibitors, although glucose supplementation had a significant effect on community composition relative to the nonsupplemented biofilms (Fig. 4) at 7 days. The addition of glucose increased the relative abundance of two groups, one represented by a 521 bp 16S DNA fragment and another represented by a 528 bp fragment, as determined by LH-PCR. LH-PCR and CV biomass determination were also performed at 14 days, when treatments had been discontinued for 6 days (two complete changes of media).

**Fig. 5:**
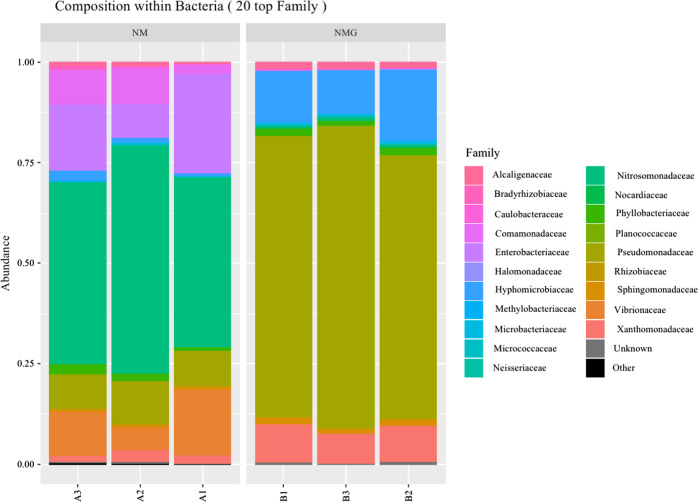
Glucose supplementation reduces community diversity of a mixed-species nitrifying community in a 4 day biofilm. Columns designated A1, A2 and A3 represent replicate bacterial cultures which were grown in nitrification medium, whereas columns designated B1, B2 and B3 represent replicate bacterial cultures which were grown in nitrification medium supplemented with 0.2% glucose (w/v). The community composition was determined by amplicon sequencing (*n* = 3), and the top 20 bacterial families detected have been represented

Some groups that were highly abundant when grown in nitrification medium without glucose were still present in the community in the presence of glucose (with and without inhibitors), such as those represented by the 518/519, 465, 468 and 500 bp fragments despite the shift in community composition. However, the increased abundance of the species represented by these fragments was associated with an overall reduction in the total diversity as determined by the Shannon–Wiener diversity index, from an average of 13 fragments per sample (*H*′ = 2.0) without glucose to eight fragments per sample when glucose was added (*H*′ = 1.6). 16S metabarcoding and amplicon sequencing indicated that the dominant organisms in the biofilms grown in nitrification medium were of the *Nitrosomonadaceae* family. *Pseudomonas* spp., *Vibrio* spp. and species from the *Enterobacteriaceae* family were also detected in lower numbers. When supplemented with glucose, *Pseudomonas* spp. became the dominant organism in biofilms, followed by *Devosia* spp. and *Vibrio*. spp., although members from the *Nitrosomonadaceae* family were still present as a minority of the total community (<1% of the total biomass) (Fig. 5).

**Fig. 6:**
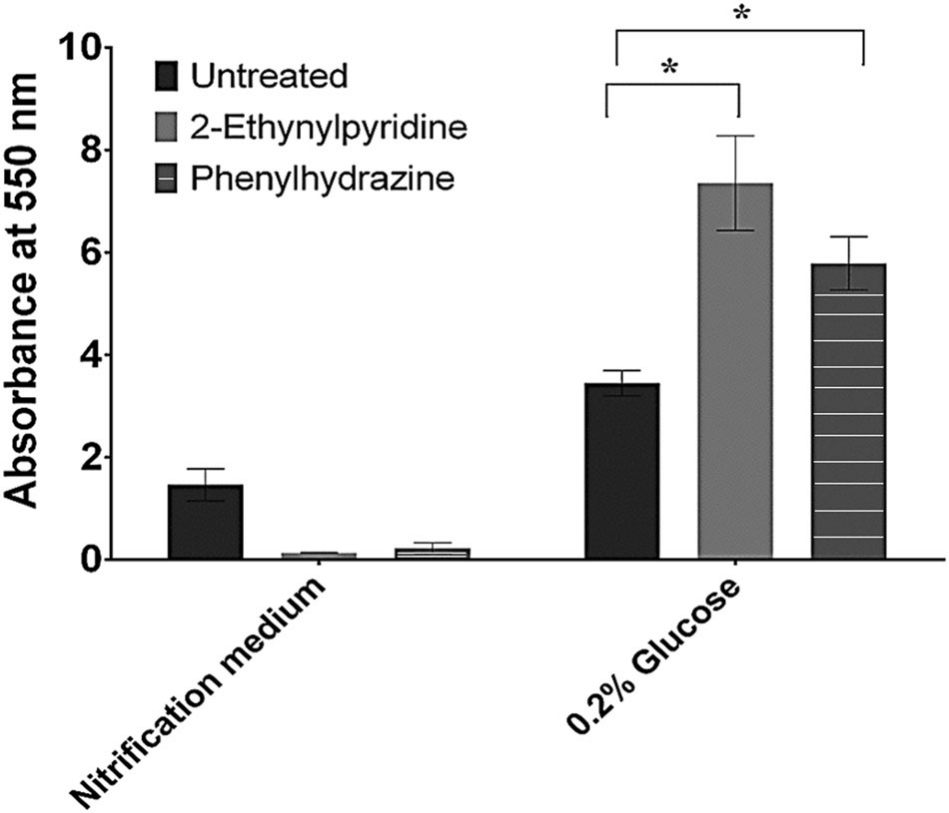
Biofilms formed by mixed-species cultures treated with glucose and nitrification inhibitors retain enhanced biomass even after discontinuation of glucose supplementation. The plates were then incubated for a further 4 days after the addition of treatment prior to the first set of measurements. The final series of treatments were therefore: **a** nitrification medium, with and without inhibitors and **b** nitrification medium with glucose, with and without inhibitors. The method followed a time line of inoculation (Supplementary Fig. S1) at 0 days (with addition of treatment, where indicated), media change at 3 days (with treatment, where indicated), media change at 7 days in untreated Nitrification medium (with biomass measurement and DNA extraction for one set, as indicated), media change with untreated nitrification medium at 10 days, and then the final biomass measurement and DNA extraction at 14 days. All measurements utilised a minimum of three biological replicate plates per treatment, with three replicate wells per plate

Almost all wells that had developed detectable biomass after 7 days of incubation continued to increase in biomass by 14 days compared to the initial 7 days measurements, as determined by increased CV staining. The inhibitor-free, glucose-treated wells were an exception, as biomass at 14 days had decreased in comparison to the initial measurements (OD_550_ decreased from ~5 to ~3.8), and biomass was not statistically different to the glucose-free, inhibitor-free biofilms (Fig. 6) (*p* > 0.05, *n* = 3). The glucose-treated, nitrification-inhibited wells were similar between inhibitor treatments (*p* > 0.05, *n* = 3), but had significantly more biomass than other treatments including the glucose-treated, non-inhibited cultures (*p* < 0.05, *n* = 3).

### The effect of glucose supplementation on pre-formed mixed-species nitrifying biofilms

The addition of glucose to a nitrifying mixed-species community at the time of inoculation resulted in a shift in reduced community diversity, as indicated by the change in Shannon–Weiner diversity index and Pielous evenness index, and a significant increase in total biofilm biomass, as indicated by the increased CV staining in Fig. 3. Therefore, it was of interest to determine the effect of glucose supplementation on pre-formed biofilms, which were biofilms that had been allowed to develop prior to the addition of treatments, in comparison with other experiments that inoculated cultures concurrently with the addition of treatments.

**Fig. 7:**
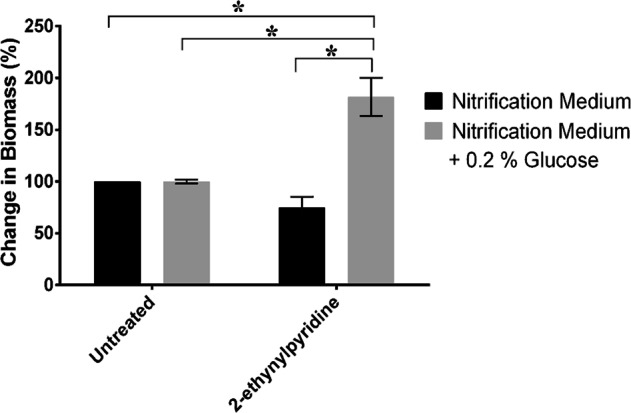
Pre-established (4 days), mixed-species nitrifying biofilms treated with glucose and 2-ethynylpyridine exhibit significantly increased biomass. Bacteria were grown in Nitrification medium for 3 days, at which point the media were replenished and the plates were further incubated for 24 h. Wells were then supplemented with 0.2% glucose (w/v), and/or 100 µM 2-ethynylpyridine and incubated for another 3 days. Data represent changes normalised against the untreated control grown in nitrification medium and are presented as the percentage change in biomass. Error bars represent the SEM (*n* = 3). Statistical significance, *p* < 0.05, is indicated by *

The biomass of biofilms which were grown for 4 days in nitrification medium prior to the addition of treatments were not affected by the addition of glucose as compared with the untreated control (Fig. 7). In contrast, treatment with 2-ethynylpyrdine reduced total biomass by 25%, although this was not statistically significant (Fig. 7). The combination of 2-ethynylpyridine and glucose supplementation significantly increased biomass by 87% compared to the untreated control (*p* < 0.05, *n* = 3).

**Fig. 8:**
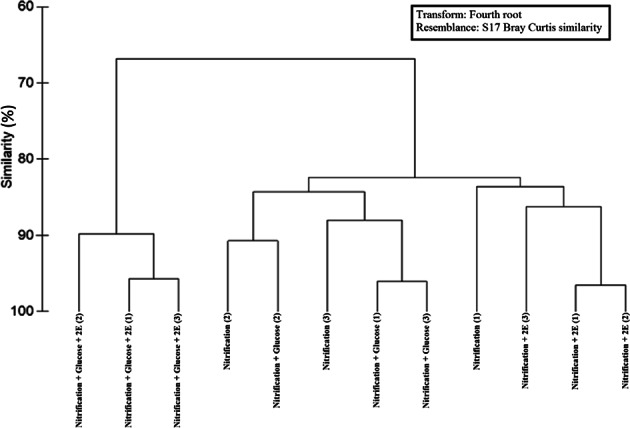
Pre-established (4 days) mixed-species nitrifying biofilms treated with glucose and 2-ethynylpyridine exhibit a shift in community composition. Bacteria were grown in Nitrification medium for 3 days, at which point the media were replenished and the plates were further incubated for 24 h. Wells were then supplemented with 0.2% glucose (w/v), and/or 100 µM 2-ethynylpyridine (2E) and incubated for another 3 days. The community composition was determined by LH-PCR and processed into a similarity table using PRIMER-6 software. Data represent the average of three technical replicates, from one biological replicate, which was representative of three biological replicates

Although the number of taxonomic groups represented by fragment sizes was unchanged, ~14 fragments, the glucose and 2-ethynylpyridine treated biofilms were significantly different to the other treatments based on the changes in fragment sizes detected as well as the spread of relative abundances (Fig. 8). They exhibited increased diversity and evenness on the Shannon–Weiner diversity index and Pielou’s evenness index (*H*′ = 2.4, *J*′ = 0.92) compared to the other biofilms (*H*′ = 2.1, *J*′ = 0.79), as well as increased biomass. The glucose and 2-ethynylpyridine treated biofilms also exhibited increased abundance of taxonomic groups represented by 16S rDNA fragments of 525, 500 and 506 bp. Interestingly, the group represented by a 528 bp fragment, which became dominant when the culture was supplemented with glucose at the time of inoculation, was only found in the pre-established biofilms when treated with both glucose and 2-ethynylpyridine.

## Discussion

The experimental system developed here represents a simple mixed-species biofilm assay that further links ammonia-oxidising bacteria with enhanced biofilm formation by heterotrophic bacteria. While the laboratory system has a number of experimental advantages, it may not completely replicate an in situ water pipeline. For example, although the biofilm was continually agitated to elicit shear stress, it was not subjected to the continuous flow of fresh water which occurs in an actual pipeline, generating greater shear stresses and delivering nutrients. It could be argued that such low flow occurs in specific regions within the pipe network, e.g., ‘dead ends’.^23^ However, this laboratory system was designed to be an experimental model that allows for the investigation of nitrifying biofilms that develop in drinking water pipelines, and to do so in relatively short experimental time frames. The experimental system described here also represents a platform upon which future work may further define the minimum culture composition from the mixed-species biofilms required to exhibit the observed effects, allowing for the development of continuous culture flow-cell modelling systems.

The concentrations of glucose used within this study (0.2%) are unlikely to be found in general drinking water distribution systems, although even greater concentrations of glucose have been observed within some agricultural drinking water systems for livestock, such as cattle^24^ and poultry.^25^ Instead of mimicking the relatively low-organic carbon concentrations of standard drinking water systems, glucose was used to stimulate the growth of heterotrophic bacteria and ensure that they were not dependent on metabolite production by ammonia-oxidising bacteria.

The results show that inhibition of nitrification activity in a mixed-species nitrifying culture disrupts biofilm formation and can disperse pre-formed biofilms, defined here as biofilms allowed to develop prior to the addition of the specified treatment. Interestingly, this dispersal effect was not observed when an organic carbon source, glucose, was included in the medium. This model suggests that AOB mediate biofilm formation by providing organic carbon that supports the growth of other organisms in media that would otherwise lack the organic carbon sources necessary for their development. Although *N*. *europaea* has previously been demonstrated to form biofilms, those biofilms generate very little biomass and have very long incubation times,^7^ demonstrating that *N*. *europaea* was a poor biofilm-forming organism. The results presented here also support that *N*. *europaea* generated very little or no biofilm when cultivated on its own.

The results presented here fit with, and add to, an earlier model^26^ suggesting that *N*. *europaea* produces organic carbon that facilitates the growth of heterotrophic organisms. Here it was shown that *N*. *europaea* is dependent on biofilm formation by those heterotrophs and hence, directly benefits from their growth. Interestingly, it was shown that supplementation with glucose resulted in a general increase in total biofilm biomass. However, this was also linked to an overall decrease in diversity and a significant loss or reduction in AOB. The biomass of established multispecies biofilms containing actively nitrifying AOBs also further increased when AOB were then inhibited via supplementation with both 2-ethynylpyridine and glucose together, in contrast to when supplemented with glucose alone. It is unlikely that the nitrification inhibitors directly influenced the growth of heterotrophic bacteria, as no change in growth was observed in the presence of glucose during these experiments. This may suggest that active AOB partially suppress or limit the overall growth of the heterotrophic bacteria. This could occur through the production of growth-limiting compounds^27^ or metabolic interference from products of ammonia oxidation, such as nitrite or hydroxylamine, which are known to be toxic or growth inhibitory.^28,29^ The concentration of nitrite produced by AOB in this study was routinely in the range of 0.69–1.73 g/L, and this may have implications for the inhibition of metabolism or induction of biofilm dispersal.^30,31^ It has previously been observed in pure cultures of *Streptococcus pneumoniae*^32^ and *Vibrio vulnificus*^33^ that the production of capsular polysaccharides restricted biofilm growth. A search of the annotated *N*. *europaea* genome revealed several genes that are potentially involved in the biosynthesis, regulation and export of capsular polysaccharides that could contribute to biofilm formation and control.^9^ Together, this indicates a role for AOB in both the formation and control of mixed-species biofilms, whereby the AOB provide organic carbon under carbon-limited conditions, while also limiting growth and expansion of those heterotrophs. Alternatively, it would be of interest to determine whether any of the present heterotrophs preferentially utilise compounds produced by AOB rather than glucose, although this is unlikely.

The data presented here suggest that the inhibition of nitrification in relatively carbon-rich systems may have the unintended consequence of enhancing biofilm formation. In systems with low concentrations of organic carbon, such as drinking water, the inhibition of nitrification disrupts a vital energy-generation pathway of ammonia oxidation to significantly reduce the potential of biofilm development and disperse pre-existing mixed-species biofilms.

By combining quantitative measures of biomass in a model multispecies biofilm with characterisation of the community composition, this study has demonstrated a potential role for AOB in the formation and control of multispecies biofilms containing HTBs, under both carbon-limited and carbon-replete conditions. This adds to an earlier study,^19^ which investigated mixed-species biofilm growth only under carbon-replete conditions that precluded the examination of the metabolic interdependence investigated here. Whereas Petrovich et al. sought to investigate the effect of adding *N*. *europaea* to established *P*. *aeruginosa* biofilms under these carbon-replete conditions, in our study, the AOB and heterotrophic bacteria were inoculated concurrently to allow for community development. This allows for better investigation of mutualistic growth and colonisation behaviour, as the initial biofilms must develop in a manner benefitting both types of organisms present. In doing so, this model better represents the development of a mixed-species community that may grow within carbon-limited conditions under natural conditions. Of particular interest is the notion that the influence of AOB is effectively dichotomous, with AOB enabling biofilm growth through the provision of organic carbon under carbon-limited conditions, at the expense of reduced total biofilm growth under carbon-replete conditions through an unknown mechanism. Such possible relationships have previously been reported.^34^ Furthermore, the results indicate that although the addition of exogenous organic carbon greatly increases the growth of heterotrophic bacteria in a biofilm (Fig. 3), AOB persist within the biofilm indefinitely and remain capable of providing lower levels of organic carbon if exogenous carbon becomes limited, as evidenced by the continued presence of biomass after the removal of glucose.

## Materials and methods

### Growth of *N. europaea* and the mixed-species nitrifying culture

AOBs were grown in NM minimal medium.^35^ The buffer solution (Solution 1) contained 54.43 g/L KH_2_PO_4_ and 4.8 g/L NaH_2_PO_4_ dissolved in deionised water to pH 8.5 using HCl and NaOH. One hundred millilitres of this buffered solution was added to 900 mL of sterile minimal salts medium (Solution 2) at final concentrations of 3.3 g/L (NH_4_)_2_SO_4_, 400 mg/L KH_2_PO_4_, 11 mg/L MgSO_4_, 22 µg/L CaCl_2_, 1.5 mg/L FeSO_4_/5 mg/L EDTA (a chelated iron mixture) and 80 ng/L CuSO_4_. Finally, a Na_2_CO_3_ solution (Solution 3) was added to the mixture to a final concentration of 40 µg/L.

A commercial nitrifying culture (Bio-Culture, AquaSonic^™^, Australia), hereafter referred to as a mixed-species nitrifying culture, or pure cultures of *N*. *europaea* (ATCC 19718) were used in this study. Based on both 16S sequencing and sequencing of the amplified *amoA* gene fragment, the dominant nitrifying organisms were of the genus, *Nitrosomonas*. For experiments with *N*. *europaea* or the mixed-species nitrifying community, nitrifying bacterial cultures were inoculated directly from glycerol stocks into 100 mL of NM in aerated Erlenmeyer flasks (500 mL), incubated in the dark at 30 °C with agitation at 100 RPM for either 14 days (mixed-species nitrifying culture) or 30 days (*N*. *europaea* pure cultures). Preliminary studies determined that this incubation time allowed the mixed-species culture to form aggregates and the measurable nitrite concentrations produced endogenously by the culture increased to 0.69–1.73 g/L (representing 10 and 25 mM, respectively), suggesting that nitrification was occurring (Supplementary Fig. 1). Maximum nitrite production was approximately 2 g/L, and in the absence of nitrification inhibitors, nitrite concentrations in these flasks were greater than 0.69 g/L for at least 60 days following inoculation. After 14 days, the bacterial culture was mixed by vortexing to more evenly distribute the floccular biomass before 1 mL was subcultured into 100 mL of fresh NM in baffled flasks to increase aeration. The flasks were then incubated for another 3 days (designated as the 3 days pre-culture), until aggregates were visible and the concentration of nitrite was between 0.69 and 1.73 g/L. The faster growth rate in the second stage of this protocol was likely the result of pre-adaptation of the nitrifying bacteria to the growth conditions.

### Growth and quantification of mixed-species and *N*. *europaea* batch biofilms

For biofilm experiments using the mixed-species nitrifying culture, biofilms were grown from a 1:100 dilution of a 3 days pre-culture in 2 mL of fresh nitrification medium into 12-well microtitre plates that were then sealed with Parafilm M^®^ (Bemis, United States), covered with aluminium foil and incubated at 25 °C with shaking (100 RPM) for 3 days. The medium was carefully removed and replaced with fresh nitrification medium and the plates were incubated for an additional 24 h, before analysing the biofilm. These conditions were determined to be optimal for the growth of mixed-species nitrifying biofilms in the absence of any treatment. The same process was used for pure cultures of *N*. *europaea*, except that the inoculum was taken from a culture incubated for 30–50 days, and the presence of aggregates was not required.

Biofilm biomass was quantified by CV staining.^36^ Briefly, the supernatant was removed and biofilms were washed once with phosphate-buffered saline (phosphate-buffered saline (PBS), 2 mL) to remove non-attached bacteria, before adding 2 mL of a 0.3 g/L CV stain solution (Becton Dickinson, United States). The plates were incubated at 25 °C for 15 min, before washing the wells twice with PBS. Finally, the CV remaining on the well’s interior surfaces was solubilised with 2 mL of 96% ethanol with 15 min of incubation at 25 °C and quantified by measuring the absorbance at 550 nm of 2 mL of the homogenised solution in a 12-well microtitre plate using a Wallac 1420 Victor^2^ spectrophotometer (PerkinElmer, United States).

### The effect of nitrification inhibition on biofilm formation and dispersal by mixed-species nitrifying biofilms

2-ethynylpyridine (Sigma-Aldrich, United States) or phenylhydrazine (Sigma-Aldrich, United States), which are known to inhibit AMO^37^ and HAO),^38^ respectively, were used to inhibit the activity of AOBs. These compounds were added to the wells from a stock solution in dimethyl sulfoxide (DMSO) (Sigma-Aldrich, United States), with a final concentration of 10.3 mg/L and 108 µg/L, representing 100 µM and 1 µM, respectively. These concentrations were experimentally determined to block nitrite production within the nitrifying culture prior to use. The compounds were added at the beginning of the experiment to investigate biofilm prevention (otherwise following the method for batch biofilm cultures, above), or after 3 d of biofilm development with incubation for a further 24 h in order to assess dispersal in developed biofilms. The biomass was then measured using CV staining.

### The effect of glucose supplementation and nitrification inhibition on biofilm formation, dispersal, and community composition by mixed-species nitrifying biofilms

To determine the effect of glucose supplementation on community composition of the mixed-species culture, a 12-well microtitre plate was prepared as shown above. Twenty microlitres of a sterile glucose solution was added to a final concentration of 0.2% (w/v), with a corresponding untreated control. After 3 days, the media was refreshed to maintain this treatment, and DNA was extracted after a further 24 h incubation (method below) for amplicon sequencing.

To determine whether there was any discrepancy in community composition between adding glucose or nitrification inhibitors to biofilms at the point of inoculation or after they had been established, 12-well microtitre plates were prepared and incubated, as indicated above, with replicates to allow for determination of biomass via CV staining and DNA extraction for LH-PCR at 7 and 14 days. Twenty microlitres of a sterile glucose solution was added to a final concentration of 0.2% (w/v), with a corresponding untreated control. This was done either at the time of inoculation for biofilm formation/inhibition assays or 3 days after inoculation for the biofilm dispersal assays. The plates were then incubated for a further 4 days after the addition of treatment prior to the first set of measurements. The final series of treatments were therefore: (a) Nitrification medium, with and without inhibitors and (b) nitrification medium with glucose, with and without inhibitors. The method followed a time line of inoculation (Supplementary Fig. S1) at 0 day (with addition of treatment, where indicated), media change at 3 day (with treatment, where indicated), media change at 7 days in untreated Nitrification medium (with biomass measurement and DNA extraction for one set, as indicated), media change with untreated nitrification medium at 10 days, and then the final biomass measurement and DNA extraction at 14 days. All measurements utilised a minimum of three biological replicate plates per treatment, with three replicate wells per plate.

### DNA extraction

DNA was extracted using the XS-hot phenol method.^39^ To extract DNA from biofilms, the supernatant was removed before the addition of 900 µL of XS buffer, 900 µL of phenol and 200 µL of sterile. After 10 min of incubation at 80 °C, the biofilm was scraped from the wells and mixed using a cell scraper and a sterile pipette tip. The plate was incubated for another 30 min before 2 mL from each well was transferred to a clean 2 mL tube and incubated at 80 °C for another 10 min. DNA was purified using phenol:chloroform:isoamyl alcohol (24:24:1), before precipitation with 100% isopropanol and 50 µL of 246 g/L sodium acetate (pH 7.5) at −20 °C overnight. DNA was resuspended in 100 µL of molecular-grade water (HyClone by GE Healthcare, United States) quantified using a NanoDrop spectrophotometer (Thermo Fisher, United States), and stored at −20 °C.

### Amplicon sequencing

Amplicon sequencing was performed for the mixed-species nitrifying community on biofilms grown with and without glucose using the facilities of the Singapore Centre for Environmental Life Sciences Engineering, at Nanyang Technological University, Singapore. Briefly, 5 ng of extracted DNA was amplified using the 515F (5′-GTGYCAGCMGCCGCGGTAA-3′) and 926R (5′-CCGYCAATTYMTTTRAGTTT-3′) primer pair with Illumina adaptors added through nested PCR, as described in the TruSeq^®^ DNA Sample Preparation Guide (Illumina, United States). Amplification consisted of an initial denaturation step of 96 °C for 3 min, followed by 30 cycles of denaturing at 96 °C for 1 min, annealing at 56 °C for 30 s and extending at 72 °C for 30 s before a final 10 min extension at 72 °C.

Amplicon sequencing was performed using the MiSeq V3 Reagent Kit and the Illumina MiSeq platform (Illumina, United States), with a read length of 2 × 300 bp. Bioinformatic analysis was conducted by stitching pair-ended reads using the Illumina Paired-End read merger tool.^40^ Reads were retained only if they contained the complete sequences of the forward and reverse primers, were ≥340 bp in length and received a *q* score of >20. Chimeras were removed from the quality trimmed reads using vsearch (v1.11.1),^41^ using both reference based (www.drive5.com/uchime/rdp_gold.fa) and de novo approaches. The dereplicated reads were clustered into OTUs at 97% of similarity using vsearch. Taxonomy was assigned to each operational taxonomic unit (OTU) using QIIME (v1.9,^42^ UCLUST method)^43^ against the Greengenes database.^44^ The table of OTUs was subsampled to 4000 sequences per sample to allow rigorous comparison between samples.

### Amplification of 16S rRNA gene and LH-PCR

The bacterial 16S rRNA V1–V3 gene region was amplified using Econo*Taq* Plus Green (Lucigen Corp., United States) to generate a ~550 bp amplicon. The reaction mix contained 50 µL Econo*Taq* Plus Green, 3.2 µM of each primer, (27 F, 5′-AGAGTTTGATCMTGGCTCAG-3′^45^ and 519R, 5′-GWATTACCGCGGCKGCTG-3′^46^), 50 ng of template DNA and molecular-grade water to a final volume of 100 µL.

For LH-PCR, the 27F primer was labelled with 6-carboxyfluorescein (6-FAM) dye (Integrated DNA Technologies, United States) at the 5′ end. The thermal cycling profile consisted of an initial denaturation step of 96 °C for 3 min, followed by 25 cycles of denaturing at 96 °C for 1 min, annealing at 55 °C for 1 min and extending at 72 °C for 45 s before a final 10 min extension at 72 °C. The product was purified using the QIAquick PCR Purification Kit (Qiagen, The Netherlands), as per the manufacturer’s instructions. LH-PCR was used in addition LH-PCR was used as it provided a cost-effective method to determine whether the variety of identified treatment combinations affected the community composition.

### Amplicon detection

The purified 6-FAM-tagged 16S rDNA amplicon from was submitted to the Ramaciotti Centre for Gene Function Analysis (UNSW Sydney, Australia) for amplicon detection using a 3730 DNA Analyser (Applied Biosystems, United States) with a LIZ600 size standard (Life Technologies, United States). Samples were analysed using PeakScanner 1.0 (Applied Biosystems, United States) to identify amplicons by comparison to the LIZ600 size standard (Thermo Fisher, United States). Amplicons outside the range of 30–600 bp were eliminated, as these fall outside of the size standards. A noise gate was applied to the orange fluorescence detection channel representing the LIZ600 standard, whereby any signal below 500 fluorescent units was considered to be noise. No noise gate was applied to the blue 6-FAM channel and all peaks with ≥1 fluorescence unit were retained. All 6-FAM peaks from the sample were then exported into T-REX (T-RFLP analysis Expedited).^47^

Briefly, the area of each peak was normalised against the combined peak area from the sample before outlying large peaks were recursively removed.^48^ This process continued until all large peaks were removed, leaving only putative background noise and generating a separate data matrix of the previously removed outliers as ‘true’ peaks. These ‘true’ peaks were then assorted into fragments sizes using T-REX in an approach modelled after T-align.^49^ The smallest peak among all samples was identified and tagged as a fragment of its given size. Any peak in the samples within the user-defined clustering threshold (in this case, 0.5 nucleotides) of this peak was then grouped into this fragment size bin representing that fragment size with a resolution of ±1 nucleotide. This process was repeated with the next-smallest peak not grouped into the first fragment size bin and continued until all peaks were grouped into such bins. Peak areas in all samples were standardised against the total peak area to output this data as relative abundance, allowing comparison between the profiles of amplicon sizes generated from LH-PCR of different samples.

### Community comparison

The fragment profiles generated from T-REX for varying treatments was analysed using the Plymouth Routines in Multivariate Ecological Research (PRIMER) statistical package (version 6.1.15) with the PERMANOVA + module (version 1.0.5) (Primer-E, New Zealand). To reduce the impact of low abundance organisms on community analysis, fragments representing less than 1% of each sample were assigned a value of 0 and any fragment sizes then not having representation in any of the samples were removed.^50^ Bacterial community structure was characterised using Bray–Curtis dissimilarity computed on fourth root transformed OTU tables. Samples were clustered by average-link clustering and represented as a dendrogram.

### Graphing and statistical analysis

General graphing and statistics were performed using Prism 6.04 graphing software (GraphPad Software, United States). For statistical analysis, one-way ANOVA was performed, followed by Bonferroni’s multiple comparisons test. Results with a *p* value of less than 0.05 were deemed statistically significant.

### Reporting summary

Further information on research design is available in the Nature Research Reporting Summary linked to this article.

## Supplementary information

Supplementary Figure S1.

Reporting Summary Checklist

## Data Availability

All of the sequence data are available through the Bioproject accession number PRJNA552789 and through the following link: https://www.ncbi.nlm.nih.gov/sra/PRJNA552789.

## Supplementary information

**Supplementary information** accompanies the paper on the *npj Biofilms and Microbiomes* website (10.1038/s41522-019-0095-4).
