# Supplementary material for: Biofilm formation inhibition and dispersal of multi-species communities containing ammonia-oxidising bacteria
Source: NPJ Biofilms Microbiomes. 2019 Aug 27;5:22. doi: 10.1038/s41522-019-0095-4 (PMC6711990; doi:10.1038/s41522-019-0095-4)
Supplement: Supplementary file 1 — Supplementary Figure S1. [file 41522_2019_95_MOESM1_ESM.pdf]

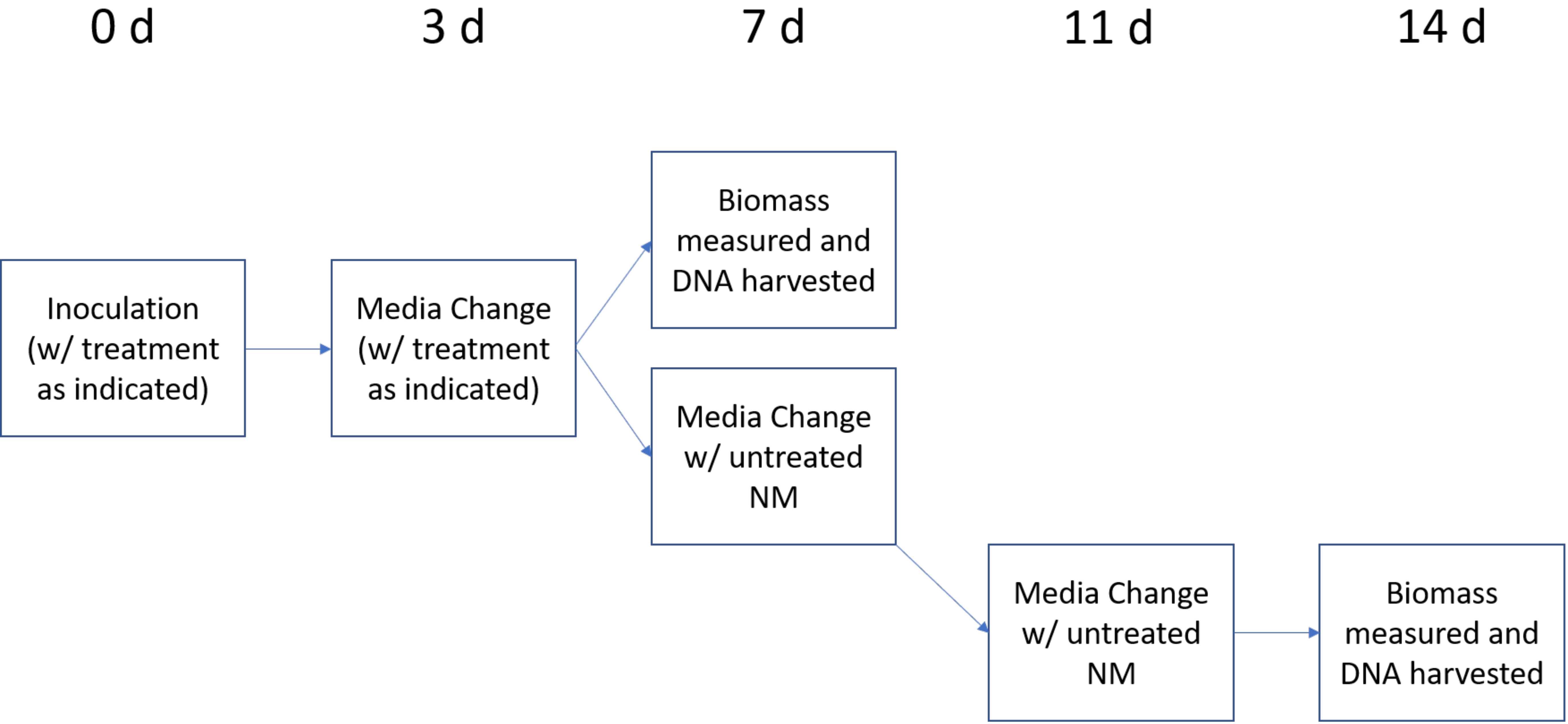

Figure S1. Flow diagram showing the steps in biofilm establishment, treatment and quantification.
